# Selected summaries

**Published:** 2008

**Authors:** B.R.J. Kannan

**Affiliations:** Department of Cardiology, Vadamalayan Hospitals, Chokkikulam, Madurai, India

**Congenital cardiac surgery without routine placement of wires for temporary pacing. Fishberger SB, Rossi AF, Bolivar JM, Lopez L, Hannan RL, Burke RP. Cardiol Young 2008;18:96-9.**

This was a retrospective study on the usage of temporary pacing wires on children operated over a 4-year period. Temporary pacing wires are valuable both for diagnosis and treatment of cardiac arrhythmias following cardiac surgery. A total of 1193 surgeries were performed between February 2002 and December 2005, excluding duct ligations and those surgeries involving pacemaker implantation as primary procedure; 762 of them were infants. The median age was 5.8 months (0 days-54 years) with a median weight of 6.2 kg (1-114 kg). The overall mortality was 2.5% with no deaths attributed to brady or tachyarrhythmia. Fourteen (1.2%) received temporary epicardial pacing wires during cardiac surgery, eight of them had high-grade atrioventricular block or sinus nodal dysfunction prior to surgery. No one needed temporary pacing after surgery. Nine (0.8%) developed postoperative junctional ectopic tachycardia and had not received the temporary pacing. They all responded to amiodarone. The data showed that the need for temporary pacing leads can be predicted preoperatively or intraoperatively.

Serious complications, including hemorrhage, tamponade, and even death, are known to occur related to postoperative temporary pacing wires. This study shows that temporary pacing was needed in a predictably small number of patients. However, this could be achieved with dedicated pediatric intensive care units with electrophysiologists available for online assessment of the rhythm problems. Improved surgical skills and myocardial protection techniques have significantly reduced the incidence of postoperative arrhythmias. The authors discussed the need for temporary pacing leads in the combined medical and surgical meetings. In addition, they reassessed the need for the pacing leads while coming off cardiopulmonary bypass coordinating with the intensive care physician. This appears to be a good approach so that routine placement of temporary pacing leads, and hence its associated complications can be avoided in large majority of children.

**Closure of symptomatic ventricular septal defects: How early is too early? Kogan B, Butler H, Kirshbom P, Kanter K, Mcconnell M. Pediatr Cardiol 2008;29:36-9.**

This is yet another study to emphasize the safety of surgery in children with large ventricular septal defects (VSDs). Between December 2002 and July 2005, 225 patients underwent closure of a VSD. The authors analyzed their data by dividing the entire cohort into four groups based on their weight. Median weights and ages at the time of surgery were 3.5 kg and 77 days (group 1), 4.9 kg and 128 days (group 2), 7.1 kg and 309 days (group 3), and 18.2 kg and 190 days (group 4). The numbers of patients were 28, 93, 47, and 57 in each of these groups, respectively. The authors analyzed all possible parameters such as cardiopulmonary bypass time, cross-clamp time, length of stay in the intensive care unit, duration of mechanical ventilation, postoperative complications, and presence of a residual VSD. There was no significant difference between the groups. There was no mortality or no persistent heart block needing pacemaker therapy.

Early surgery for VSD is now an established practice in most of the pediatric cardiac centers, and hence the result of this study is not surprising given the vast experience of health personnel dealing with even smaller children with more complicated heart diseases. The authors clearly showed that the duration of the operation or the postoperative complications are not influenced by weight or the age of children. However, it is surprising to note that even in a center from the developed country, surgical closure of a simple VSD is not recommended until the child attains a weight of 5 kg or an age of 5 months. Till such time, the authors have reiterated the minimal role of medical therapy.

**Implantation of stents for treatment of recurrent and native coarctation in children weighing less than 20 kilograms. Schaeffler R, Kolax T, Hesse C, Peuster M. Cardiol Young 2007;17:617-22.**

Majority of aortic coarctations presenting beyond infancy do not involve the arch. Hence, there has been a constant debate regarding the initial mode of therapy, surgical vs. transcatheter. This small study involved nine patients who underwent stenting of coarctation. Eight were recurrent coarcation of which seven had recoactation at a mean interval of 42 months after surgery and one presented with restenosis 6 months after balloon dilatation. Two of them weighed 6 kg and the others weighed ≥16 kg. The small children received Palmaz Genesis (124P) and JoStent through 5 F and 6 F arterial sheaths, respectively, and the others received Palmaz Genesis (1910XD) through 7 or 8 F sheaths. The procedure was successful in all and the mean peak systolic gradient reduced from 30 to 3 mmHg. There was no complication except for temporary weakness of lower limb pulses in a child, which responded to heparin. At a mean follow-up of 15.8 months, redilatation was needed for a child, 24 months after the procedure. No stent fracture or aneurysm was noted.

While surgical treatment of coarctation of aorta has consistently shown a reduction in the need for reintervention or the risk of late complications, patients can have long-term issues related to thoracotomy and its effect on the lung. Transcatheter stent implantations need large delivery sheaths, and hence it has not been preferred in smaller children. The authors have shown the safety of this procedure in children weighing ≤20 kg. However, only two of them weighed ≤6 kg. As the stents do not grow, repeat dilatations are mandatory if implanted in small children. Within a short follow-up period of 2 years, one of them needed a redilatation and repeat procedure was scheduled in two others. This study is obviously limited by its small size and short follow-up.

**The Amplatzer Membranous VSD Occluder and the vulnerability of the atrioventricular conduction system. Fischer G, Apostolopoulou SC, Rammos S, Schneider MB, Bjørnstad PG, Kramer HH. Cardiol Young 2007;17:499-504.**

The authors have reported their experience in closing the perimembranous ventricular septal defects (VSD) in 35 patients using an amplatzer membranous VSD occluder. They chose patients with evidence of left ventricular volume overload with VSD having an aortic rim of ≥2 mm. The median weight of their patient population was 16 kg. The median ratio of pulmonary to systemic flows was 1.9 with the median fluoroscopic time of 27 min. Six patients had membranous septal aneurysm and two of them needed balloon dilatation to allow the passage of the delivery sheath. All were closed using the amplatzer perimembranous VSD occluder except in three where muscular VSD occluder was used as they had 4-7 mm tissue rim from the aortic valve. One patient developed aortic regurgitation needing surgical removal of the device and closure of the defect. Trivial or mild new aortic regurgitation was seen in nine, which disappeared in seven subsequently. Complete closure was achieved in 53% at 1 month and 91% at 2 years. One patient had moderate residual shunt and underwent surgical closure 2 years later. In the rest, residual shunts were small and showed resolution of the left ventricular enlargement. One patient had transient left bundle branch block, whereas six others (17%) had permanent conduction disturbances. Two had right bundle branch block, two had left anterior hemiblock and one had bifascicular block. One patient with left bundle branch block progressed to Mobitz type II block 6 months after implantation and in another child complete heart block developed 11 months after the procedure. Two patients had ventricular couplets 24 h later with spontaneous resolution.

It is commendable that the authors performed the closure of perimembranous VSD with a relatively short fluoroscopic time. They have shown the feasibility of this procedure with the amplatzer device, specifically designed for this. Although the complications related to tricuspid valve was negligible even in those with septal aneurysm formation, the incidence of aortic regurgitation and the immediate and late development of conduction disturbances is quite alarming. The authors have justified the indication for closure of these defects based on volume overload of left ventricle. Although they have equated with the late complications related to surgical closure with the complications related to the device closure, the patient populations were different with symptomatic younger children with large VSD treated with surgery where the benefits outweighed the risks.

**Transcatheter closure of atrial septal defect without balloon sizing. Wang J, Tsai S, Lin S, Chiu S, Lin M, Wu M. Catheter Cardiovasc Interv 2008:71:214-21.**

The authors attempted device closure of atrial septal defects without balloon sizing in 243 patients over a 27- month period. The age of the patients ranged between 2.1 and 76 with a median of 22 years. They compared the results with 271 patients who had earlier undergone device closure based on balloon sizing. Initially, the authors chose a device 4-6 mm larger if the ASD size assessed by transesophageal echocardiography was < 14 mm and 6-8 mm larger if the ASD measured ≥14 mm in diameter. In the later part of their study, they selected slightly smaller devices, 4-5 and 5-7 mm for < 14 and ≥14 mm defects, respectively. A total of 247 devices were deployed in 240 patients. The median defect diameter was 17 mm (5-37). The mean device to defect diameter ratio was 1.4 and 1.3, if maximal defect diameter was < 14 and >14 mm, respectively. Three patients needed a larger device than that chosen initially while two others needed a smaller device in view of impingement on the mitral valve. Device embolization occurred in two patients. Compared to balloon sizing group, there was no difference in the success rate, complications or incidence of late complete closure.

With growing experience and more clear understanding of the atrial septal defects by imaging studies, there has been a tendency to avoid balloon sizing of the ASD to choose the appropriate device. While the previous studies used the firm edges of the atrial septal defect or the width of the color flow to choose the device size, these authors arbitrarily chose a device larger by 5-8 mm than the size measured by echocardiography. The basis of how this formula was derived has not been explained. Avoiding balloon sizing can reduce the procedural cost, radiation exposure and possible complications related to prolonged inflation. The size of the device selected by these authors roughly corresponded to the size chosen by others based on balloon sizing. Hence, this method of choosing the device size did not result in deployment of smaller devices as compared to others.

